# Effects of Alkali Stress on the Growth and Menaquinone-7 Metabolism of *Bacillus subtilis natto*

**DOI:** 10.3389/fmicb.2022.899802

**Published:** 2022-04-28

**Authors:** Xiaoqian Chen, Chao Shang, Huimin Zhang, Cuicui Sun, Guofang Zhang, Libo Liu, Chun Li, Aili Li, Peng Du

**Affiliations:** ^1^Key Laboratory of Dairy Science, College of Food Science, Northeast Agricultural University, Harbin, China; ^2^Heilongjiang Green Food Science Research Institute, Harbin, China

**Keywords:** menaquinone-7, *Bacillus subtilis natto*, microbial fermentation, alkali stress, transcriptome

## Abstract

Menaquinone-7 (MK-7) is an important vitamin K_2_, synthesized from the menaquinone parent ring and seven isoprene side chains. Presently, the synthesis of MK-7 stimulated by environmental stress primarily focuses on oxygen stress, while the effect of alkali stress is rarely studied. Therefore, this study researched the effects of alkali stress on the fermentation performance and gene expression of *Bacillus subtilis natto*. The organism’s growth characteristics, biomass, sporogenesis, MK-7 biosynthesis, and gene expression were analyzed. After a pH 8.5 stress adaptation treatment for 0.5 h and subsequent fermentation at pH 8.5, which promoted the growth of the strain and inhibited the spore formation rate. In addition, biomass was significantly increased (*P* < 0.05). The conversion rate of glycerol to MK-7 was 1.68 times higher than that of the control group, and the yield of MK-7 increased to 2.10 times. Transcriptomic analysis showed that the MK-7 high-yielding strain had enhanced carbon source utilization, increased glycerol and pyruvate metabolism, enhanced the Embden-Meyerhof pathway (EMP), tricarboxylic acid (TCA) circulation flux, and terpenoid biosynthesis pathway, and promoted the accumulation of acetyl-CoA, the side-chain precursor of isoprene. At the same time, the up-regulation of transketolase increased the metabolic flux of the pentose phosphate (HMP) pathway, which was conducive to the accumulation of D-erythrose 4-phosphate, the precursor of the menadione parent ring. This study’s results contribute to a better understanding of the effects of environmental stress on MK-7 fermentation by *Bacillus subtilis natto* and the molecular regulatory mechanism of MK-7 biosynthesis.

## Introduction

Menaquinone-7 (MK-7) is a type of vitamin K_2_ composed of a mother ring of menadione and seven isoprene side chains. MK-7 plays a role in respiratory chain transmission, coagulation function, and calcium homeostasis (Turck et al., 2017; Wilkens et al., 2021; Wu et al., 2021). In addition, MK-7 has more and more benefits in promoting bone healing, preventing cerebrovascular and cardiovascular diseases and fighting cancer cells, Alzheimer’s disease and Parkinson’s disease (Halder et al., 2019; Mahdinia et al., 2019a; Tarkesh et al., 2020). However, the human body cannot synthesize MK-7, and the only channels to obtain it are food and dietary supplements (Bergeland et al., 2019; Brudzynski and Flick, 2019).

The content of MK-7 from natural food sources is too low to obtain preventive and therapeutic doses, such as pork liver oil, cheese and certain vegetables (kale and celery; Mahdinia et al., 2017). Therefore, it is mainly through nutritional supplements to achieve biomedical-level applications. Chemical synthesis has been the most common method of MK-7 production in the past (Łaszcz et al., 2018). However, chemical precursors for synthesis are of limited origin and produce both *cis* and *trans* isomers (Baj et al., 2016; Brudzynski and Maldonado-Alvarez, 2018; Vermeer et al., 2018), with only the latter being biologically active. So, MK-7 obtained by chemical synthesis has low biological activity and is prone to causing environmental pollution. In recent years, researchers have developed a biological fermentation method for the synthesis of MK-7 to obtain a more natural and active form of vitamin K_2_ (Zhao et al., 2021; Bus et al., 2022), of which MK-7 accounts for about 97% (Mahdinia et al., 2019b; Zhao et al., 2021).

Menaquinone-7 contains some special structures, such as isoprene side chains and quinone skeleton, leading most bacteria to synthesize MK-7 in a complex way, increasing energy consumption and production costs. Hence, raising MK-7 production and reducing production costs is still a difficult problem (Berenjian et al., 2015). The key problems to be solved at present are to screen MK-7 producing strains efficiently and optimize fermentation conditions. Many researchers have used breeding (Song et al., 2014; Xu and Zhang, 2017), genetic modification (Liu et al., 2019; Yang et al., 2019), and optimized fermentation processes (Novin et al., 2020) to increase the yield of MK-7. With the development of metabolic (Xu et al., 2017; Chen T. et al., 2020) and genetic engineering technologies (Cui et al., 2019), related biotechnologies have been extensively studied to regulation MK-7 biosynthesis, while the study of abiotic stress methods to increase MK-7 production limited. *B. subtilis natto* is not only the main microorganism in the industrial production of MK-7, but also an excellent production source, which can be secreted intracellularly and extracellularly (Shih et al., 2005). In addition, the strain has some advantages, such as the high yield of MK-7, easy growth and culture, short fermentation time, resistance to environmental stress, and so on (Wu, 2019). Studies have found that environmental stress can induce changes in the metabolism of probiotics and secrete metabolites beneficial to their survival (Li et al., 2016). Our previous study confirmed that environmental stress can stimulate the production of metabolites and improve the stress tolerance of lactic acid bacteria (Chen X. et al., 2020; Zhang et al., 2020). In addition, Zeng et al. (2014) showed that heat stress promoted the synthesis of poly (γ-glutamic acid) and its precursors by *B. subtilis* GXA-28. And salt stress improved the poly (γ-glutamic acid) synthesis ability of *Bacillus licheniformis* WX-02 (Wei et al., 2010).

The omics technique is a method to explain how microorganisms respond to environmental changes at the level of molecular mechanism. Currently, technologies such as metabolomics, proteomics and transcriptomics have been used to explore the mechanisms of MK-7 metabolic changes under environmental stress (Priyadarshi et al., 2009; Ma Y. et al., 2019). Ma Y. et al. (2019) have found that under the condition of high oxygen supply at 200 rpm, the yield of MK-7 could be doubled through transcriptome research. Xu et al. (2017) found that overexpression of key enzymes in the metabolic pathway resulted in a 1.6-fold increase in MK-7 content through metabolomics studies. Therefore, omics technology can be applied to study the molecular mechanism of *B. subtilis natto* under environmental stress.

In this study, the aim was to study the effects of salt, acid, alkali, and temperature stress on the growth and MK-7 biosynthesis of *B. subtilis natto*, and analyze the optimal stress conditions to improve the biosynthesis of MK-7. In addition, transcriptome was used to study the effect of alkali stress on gene expression in the MK-7 enrichment pathway of *B. subtilis natto*. This study could provide a new perspective for extending techniques to increase MK-7 production and deepen understanding of the molecular mechanisms underlying MK-7 biosynthesis.

## Materials and Methods

### Strain Culture Condition

The experimental strain was *B. subtilis natto* CICC10262. The cultivation conditions and methods were the same as described by Berenjian et al. (2011). The formula of the fermentation medium was glycerol 50 g/L, soybean peptone 150 g/L, yeast extract 50 g/L, K_2_HPO_4_ 0.6 g/L. The passage medium and fermentation culture were carried out at speeds of 170 rpm and 200 rpm, respectively. Furthermore, we studied some of the intracellular reactions.

In order to determine the exact value of the optimal pH of *B. subtilis natto* CICC10262 growth medium, 3% (v/v) of seed culture in the exponential phase (OD_600_ ∼ 0.8 or 10^8^ CFU/mL) have been inoculated to 14 cultures with different initial pH (The pH of the medium was set to 3, 4, 4.5, 5, 5.5, 6, 6.5, 7, 7.5, 8, 8.5, 9, 9.5, and 10, respectively), and the number of viable bacteria was then monitored. All cultures were performed under aseptic conditions. The shaker incubator parameters were set to 170 rpm and 37°C.

### Determination of Biomass and pH

Biomass and pH measurements have been slightly modified as described by Rincón Santamaría et al. (2019). The biomass was measured at 600 nm using a multifunctional enzyme reader [SpectraMax i3X, Molecular Devices (Shanghai, China) Co., Ltd.]. The pH-meter was calibrated at 25°C.

### Determination of Adaptation Conditions to Stress

The activated thalli were inoculated into nutrient agar (NA) medium with 3% (v/v) inoculum for 10 h and cultured in NA medium, then 20 mL of bacterial solution was taken, centrifuged at 8000 rpm for 10 min to collect thalli. The cells were suspended in equal volumes of NA medium containing 1% (w/v), 3% (w/v), and 5% (w/v) NaCl and pH 4.0, 4.5, and 5.0, and incubated at 37°C for 0.5 h. In addition, incubated at 42°C, 45°C, and 48°C for 0.5 h to carry out high-temperature adaptation treatment. After the stress adaptation, the bacteria collected after treatment were washed twice with physiological saline, and the bacteria were centrifuged at 8000 rpm for 10 min to collect thalli. The thalli were suspended in 20 mL NA liquid medium, shaken well, and inoculated in NA medium with 3% (v/v) for 24 h, and the colonies were counted to determine the best suitable treatment conditions.

### Determination of Number of Viable Bacteria

The number of viable bacteria in fermentation broth was determined by the dilution coating plate method described by Li et al. (2021). To measure the number of viable bacteria, each sample was diluted 10 times with physiological saline, and then the 100 μL diluted solution was coated on the nutrient agar plates in triplicate. The plates were incubated at 37°C for 24 h. The number of colonies on each plate ranged from 30 to 300, and the results were expressed in logarithmic form.

### Determination of Residual Glycerol Analysis

Carbon source can affect the growth and metabolism of microorganisms, and glycerin has been proved to promote the production of MK-7 by *B. subtilis natto*, so glycerin was selected as the carbon source of this experiment. Carbon source content in fermentation broth was determined by the method described by Zhou et al. (2016), that was, the titration method was used to detect changes in glycerol concentration. Centrifuged 2.0 mL of fermentation broth at 8000 rpm for 3 min, sucked 1.0 mL of supernatant, added 10 mL of distilled water, added 2 drops of phenolphthalein as an indicator, and fixed with 0.05 mol/L NaOH standard drops until color changes. Recorded the NaOH dosage V_1_. Then added 10 mL of 0.1 mol/L sodium periodate solution, reacted for 5 min in the absence of light, and then added 5.0 mL of 25% ethylene glycol, and continued to react in the absence of light for 5 min. Then used NaOH standard drops were used to fix to change color, recorded the amount of NaOH as V_2_ at this time, and the formula was as follows.

ω⁢(Glycerol)=92.1⁢c×(V2-V⁢1)/M×100%

Among:

V_1_: The volume of the NaOH standard solution used for the blank test, mL;

V_2_: The volume of the NaOH standard solution used for the determination of sample, mL;

C: Concentration of the NaOH standard solution used, mol/L;

M: Mass of test sample, g;

92.1: Molar mass of glycerol, g/mol.

### Determination of Spore Formation Rate

The sporulation rate of *B. subtilis natto* during fermentation was measured as described by Luo et al. (2016a). The cells were stained with ammonium oxalate crystal violet for 1 ∼ 2 min, washed to remove the floating color, and then randomly selected 5 fields under 100 × multiple oil microscope to count the number of transparent spores and the total number of cells. Detection was performed at the end of fermentation. The formula for calculating the sporulation rate was as follows.

Sporulationrate/%=(Number⁢of⁢spores)/(Total⁢number⁢of⁢cells)×100

### Menaquinone-7 Extraction and Analysis

The extraction and content of MK-7 in fermentation broth were measured as described by Sun et al. (2020). In our preliminary studies, *n*-hexane:2-propanol (2:1, v/v) with 1:4 (liquid: organic, v/v) was found to be the optimal mixture for MK-7 extractions. During each extraction, the mixture was shaken vigorously with a vortex mixer for 2 min and centrifuged at 3000 rpm for 10 min to separate two phases. The organic phase was then vaporized in the vacuum, and the extracted MK-7 was eventually recovered. High performance liquid chromatography (HPLC) Waters 2695-2420 (Nanchang Jiedao Scientific Instruments Co., Ltd., China) equipped with a UV detector and a C18 chromatographic column (5 μm, 250 × 4.6 mm, Hypersil ODS2, China), for analysis of MK-7 concentration at 40°C. Methanol: Dichloromethane (9:1, v/v) was the mobile phase at a flow rate of 1.0 mL/min and a wavelength of 254 nm for calibration and analysis.

### RNA Isolation, Library Construction, and Sequencing

The fermentation broth was collected from the control group and the alkali stress group (pH 8.5), respectively. Immediately centrifuged at 8000 rpm for 10 min, stored at −80°C, and extracted with RNA lock Reagent. The specific procedures for RNA separation and sequencing were slightly modified by referring to Cheng et al. (2020).

### RNA–Seq Data Analyses

Clean data was mapped to the reference sequence as described by Langmead and Salzberg (2012). The expression levels of genes and transcripts were estimated according to the method of Robinson et al. (2010). A mathematical statistical model was used to identify differentially expressed genes (DEG) in the control group and the alkali stress group as described by Kong et al. (2007). Finally, the DEGs were determined by the Log_2_ ratio of ≥1 and FDR of ≤0.05. Then they were mapped to gene ontology (GO) and KEGG database for the functional and pathway analysis.

### Statistical Analysis

Three biological replicates were performed, and three technical replicates were performed in each group. Data were analyzed using SPSS 20.0 (IBM SPSS Statistics, IBM Corp., Somers, NY, United States) for one-way analysis of variance (ANOVA). Results with *P* < 0.05 were considered statistically significant.

## Results and Discussion

### The Optimum Growth and Fermentation Conditions of *Bacillus subtilis natto* CICC10262

**FIGURE 1:**
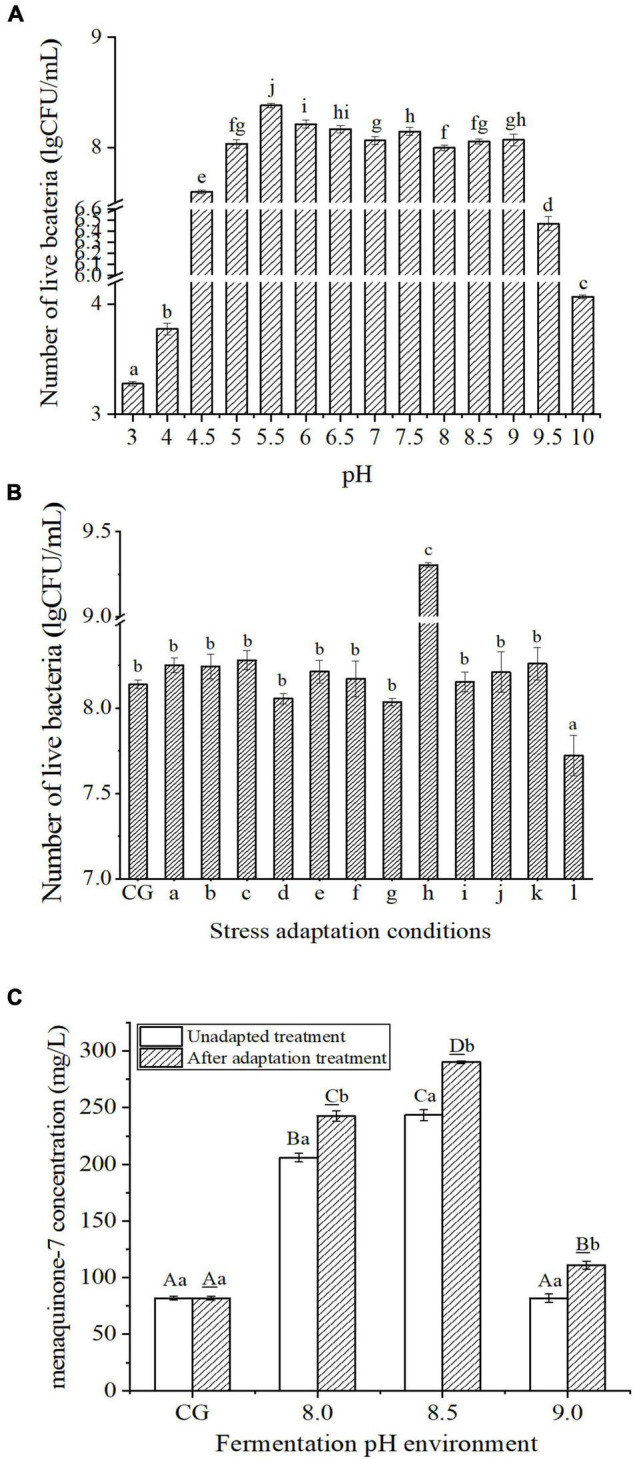
**(A)** Growth of *Bacillus subtilis natto* CICC10262 at different pH. Different lowercase letters indicated that the values of each group were significantly different, *P* < 0.05. **(B)** Effects of different stress adaptation conditions on the growth of *Bacillus subtilis natto* CICC 10262. In group a, b, and c indicated that the strain was adapted to (nutrient agar) NA medium containing 1% (w/v), 3% (w/v), and 5% (w/v) NaCl for 0.5 h, d, e, f, g, h, i were strains adapted to NA medium at pH 4.5, 5.0, 6.0, 8.0, 8.5, 9.0 for 0.5 h, j, k, l were strains incubated at 42°C, 45°C, 48°C for 0.5 h, then inoculated into NA medium containing 3% (w/v) NaCl with normal pH and cultured at 37°C for 24 h. In the significance analysis, different lowercase letters indicated that the values of each group were significantly different, *P* < 0.05. **(C)** The yield of MK-7 fermented by strain under different pH conditions. Different lowercase letters indicated that there were significant differences between different stress intensities in the unadapted treatment group, *P* < 0.05. Different capital letters indicate that under the same pH condition, the difference between the unadapted treatment group and the adaptation treatment group is statistically significant, *P* < 0.05.

The number of live bacteria of *B. subtilis natto* CICC10262 in the different treated groups is shown in Figure 1A. The optimum pH range for the growth of *B. subtilis natto* was wide, 5.0 ∼ 9.0, which was consistent with the findings of Luo et al. (2016b). However, after growing at pH 5.5 for 24 h, the number of viable bacteria was the highest, supporting the conclusion of Yang and Zhang (2011) that *Bacillus subtilis* grows vigorously in weak acid and neutral environments.

Figure 1B shows the number of viable *B. subtilis natto* after different stress adaptation treatments for 0.5 h and subsequent culturing in nutrient agar (NA) medium for 24 h at normal pH (the pH was not adjusted after sterilization and was between 5.5 and 6.0). Compared with the control group (without adaptation treatment), the number of viable bacteria in the treated group significantly decreased by 9.63% after incubation at 48°C for 0.5 h (*P* < 0.05), there was no significant change in the number of viable bacteria after other treatment. The number of viable bacteria increased significantly and reached the maximum after culturing for 0.5 h in NA medium with a pH 8.5 (*P* < 0.05), which was 1.14 times higher than that of the control group. Therefore, pH 8.5 adapted to 0.5 h was found to be the best adaptation treatment condition.

To study the effect of *B. subtilis natto* on the biosynthesis of MK-7 after the adaptation treatment, it was cultured for 0.5 h at pH 8.5. The treated bacteria were then placed into a normal beef extract peptone medium and cultured to the end of the logarithmic growth phase. After that, *B. subtilis natto* was inoculated into different alkaline pH fermentation mediums. The effects of the different alkaline pH conditions on the yield of MK-7 are shown in Figure 1C. Compared with untreated *B. subtilis natto*, the yield of MK-7 was significantly increased by the stress adaptation treatment and fermentation under alkali stress (*P* < 0.05). After stress adaptation treatment, the yield of MK-7 was highest at pH 8.5 (290.19 ± 1.12 mg/L), which was 2.10 times that of the control group. The results showed that the production of MK-7 could be significantly promoted by the alkaline environment of the fermentation medium and stress adaptation treatment of *B. subtilis natto* during its growth stage.

### Fermentation Process of *Bacillus subtilis natto* CICC10262

**FIGURE 2:**
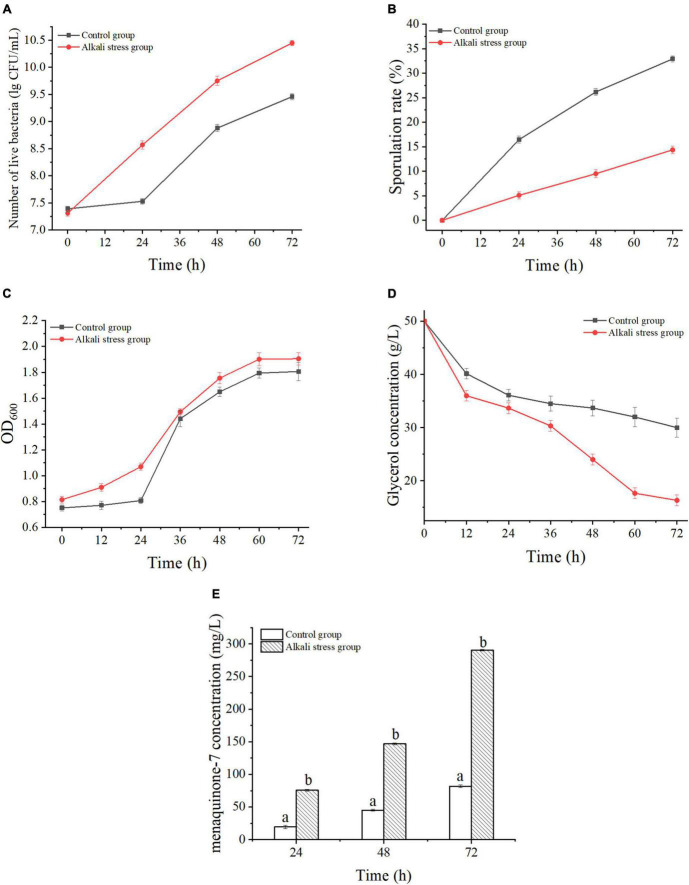
Fermentation characteristics of *Bacillus subtilis natto* CICC10262 under alkali stress. **(A)** Live bacteria, **(B)** Sporulation rate, **(C)** Cellular biomass (OD_600_), **(D)** Glycerol consumption, **(E)** MK-7 yield. Differences in lowercase letters indicate significant differences between the two groups of values at the same time, *P* < 0.05.

Two different fermentation conditions were designed to understand the changes of the MK-7 biosynthesis process of *B. subtilis natto* after adaptation treatment and alkali stress. One fermentation medium had a normal pH was set as the control group; the other medium’s pH was adjusted to pH 8.5. The changes in the number of live bacteria, sporulation rate, biomass, glycerol consumption, and MK-7 biosynthesis using the two different fermentation mediums are shown in Figure 2. After the *B. subtilis natto* was adapted to alkali stress, its growth was promoted, and the number of viable bacteria was 1.43 times that of the control group (Figure 2A); simultaneously, these conditions had an inhibitory effect on the rate of spore formation, and the spore rate decreased significantly by 56.32% (*P* < 0.05) (Figure 2B). Furthermore, the biomass in the two fermentation mediums entered the stable phase at 60 h. However, the biomass of the alkali stress group was always 3.68∼5.48% higher than that of the control group, indicating that the alkali stress adaptation treatment enhanced the biomass significantly (*P* < 0.05) (Figure 2C). The glycerol consumption under alkali stress conditions was faster than that of the control group, and the conversion rate of glycerol to MK-7 was 1.68 times that of the control group (Figure 2D). As MK-7 is a secondary metabolite and accumulates at the late stage of fermentation, significant accumulation of MK-7 was detected after 24 h. In addition, the yield of MK-7 was higher than that of the control group at all time points of fermentation after alkali stress treatment. The maximum yield was 290.19 ± 1.12 mg/L, which was 2.10 times that of the control group (Figure 2E). And, as shown in Figures 2D,E, the concentration of MK-7 in cells under alkali stress improved significantly with increasing consumption of glycerol as a carbon source and was positively correlated. These results were similar to those of previous studies: the substrate consumption increased under certain conditions of alkali stress and thus promoted the increase of metabolites (Ma Y. et al., 2019; Cheng et al., 2020). In addition, to evaluate the fermentation capacity of *B. subtilis natto* to produce MK-7, we studied the productivity change of MK-7. At the initial stage of fermentation, there was a significant difference in MK-7 yield between the two fermentation conditions (*P* < 0.05) and the difference continued to increase with the prolongation of fermentation time. Combined with Figure 2A, the alkali stress treatment increased the growth of *B. subtilis natto* by 12% compared to the control group. Therefore, alkali stress treatment could enhance the biosynthesis ability of *B. subtilis natto* to produce MK-7.

### Transcriptome Analysis of Menaquinone-7 Production Gene Expression in *Bacillus subtilis natto* CICC1026

**FIGURE 3:**
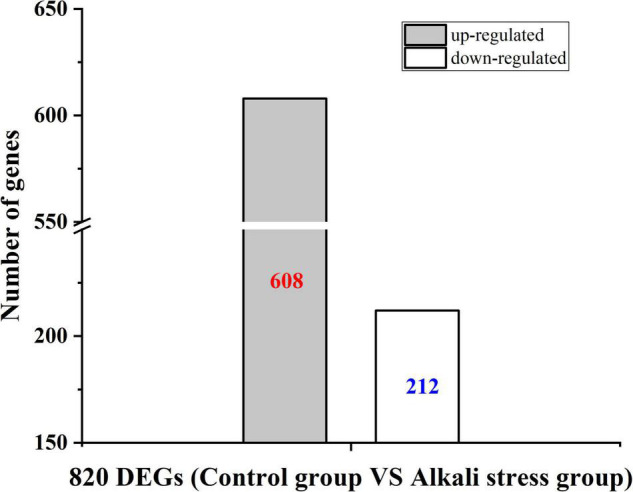
Differential gene expression of control strain and strain after adaptation treatment in the fermentation process under alkali stress.

To better understand how alkali stress affects *B. subtilis natto* CICC 10262’s production of MK-7, the differences in gene expression in the metabolic process under alkali stress were compared using transcriptome analysis. We identified a total of 820 differently expressed genes (DEGs) (Figure 3). Among these genes, 608 were found to be up-regulated and 212 down-regulated.

#### Gene Ontology and Kyoto Encyclopedia of Genes and Genomes Database Analysis

**FIGURE 4:**
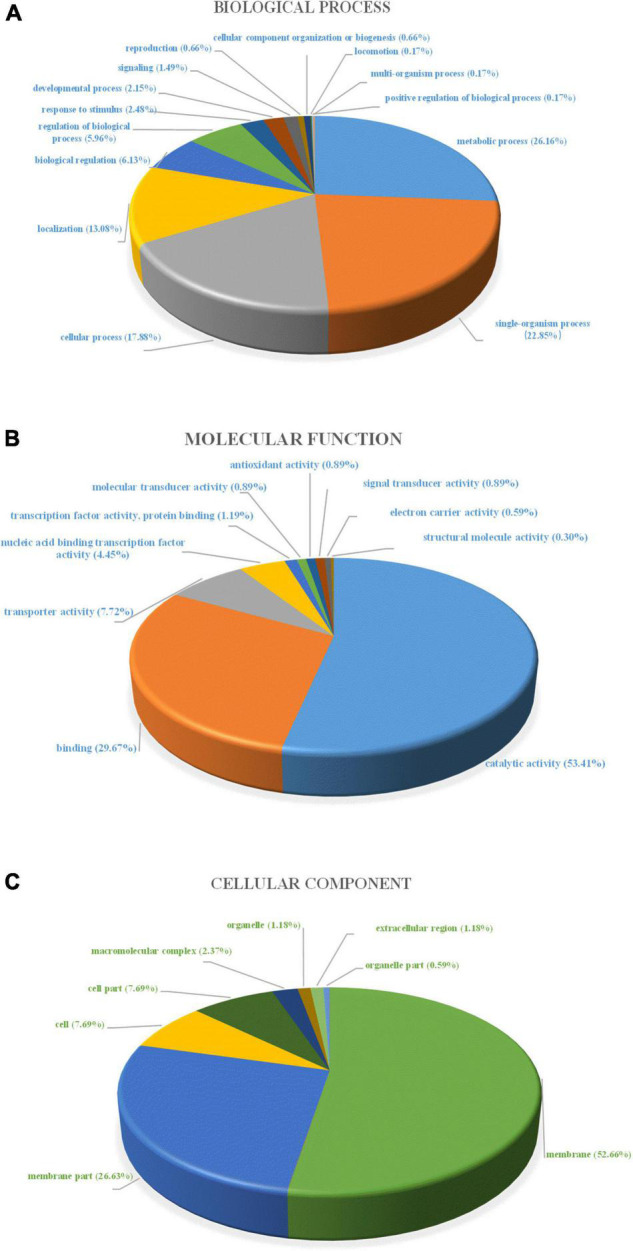
Gene ontology (GO) functional analysis of unique sequences from control group and alkali stress group transcriptome. Unique sequences were assigned to three categories: **(A)** Biological process, **(B)** Molecular function, **(C)** Cellular component.

**FIGURE 5:**
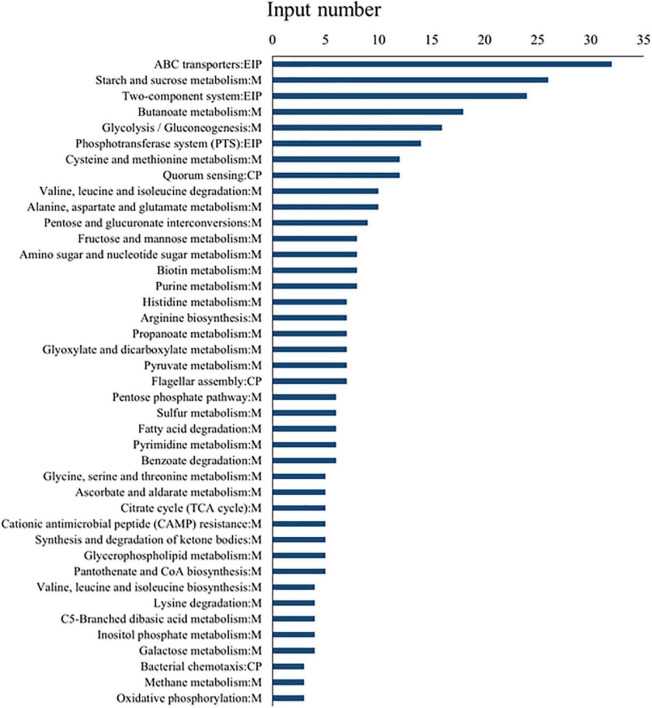
KEGG pathway enrichment analysis of differential genes. Among them, EIP represents environmental information processing, M represents metabolism, and CP represents cellular processes.

To understand the functional information of differential genes, Gene Ontology (GO) and Kyoto Encyclopedia of Genes and Genomes (KEGG) databases were used for gene annotation and functional classification of predicted protein-coding genes (CDSs). In Figure 4, we summarize three GO functional categories, including *biological processes*, *cellular component*, and *molecular function*. Each category was annotated with 3,605, 1,031, and 2,106 genes, respectively. We found a large number of genes encoding catalytic activity (1035 genes), metabolic (989 genes), cellular (801 genes), single-organism (731 genes), and localization (325 genes) processes, and binding (673 genes), and membrane (460 genes) function. The KEGG database analysis revealed that these DEGs existed in three different enrichment pathways: cellular processes, environmental information processing, and metabolism. Of these pathways, metabolic changes were the most significant (*P* < 0.05) (Figure 5). A total of 1,679 CDSs were annotated to 65 KEGG functional categories, of which “carbohydrate metabolism” accounted for the largest proportion (24.36%, 409 genes) in the secondary classification, followed by “amino acid metabolism” (14.89%, 250 genes), “membrane transport” (9.77%, 164 genes), and “metabolism of cofactors and vitamins” (8.40%, 141 genes), as can be seen in Figure 5. These results show that the functions of the CDSs are mainly the metabolism and transportation of substances, which provide energy for the life activities of *B. subtilis natto* and also provide precursors for the synthesis of secondary metabolites (Wang et al., 2018). Explicitly, it included ATP-binding cassette (ABC) transporters, phosphotransferase system (PTS), purine, amino acid, glycerol and ketone body metabolism, peroxisome, and glycolysis/gluconeogenesis, and so on. The ABC transporters and PTS pathways were related to membrane transport (Zhou et al., 2020), which may affect the secretion of MK-7. These DEGs may be involved in alkali stress and MK-7 production.

#### Differentially Expressed Genes Related to Menaquinone-7 Biosynthesis

**FIGURE 6:**
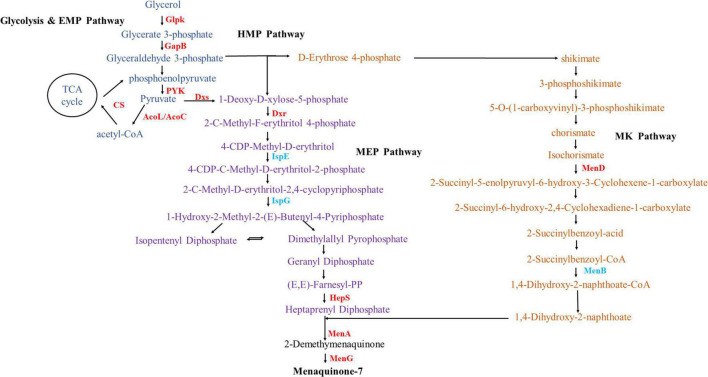
Changes in transcript abundance of enzymes involved in menaquinone-7 metabolic pathway. Enzymes: GlpK, glycerol kinase; GapB, glyceraldehyde-3-phosphate dehydrogenase (NADP-dependent, gluconeogenesis); CS, citrate synthase; AcoL/AcoC, acetoin dehydrogenase E3/E2 component (dihydrolipoamide dehydrogenase); Dxs, 1-deoxyxylulose-5-phosphate synthase; Dxr, 1-deoxy-D-xylulose 5-phosphate reductoisomerase; IspE, 4-diphosphocytidyl-2-C-methyl-D-erythritol kinase; IspG, 4-hydroxy-3-methylbut-2-en-1-yl diphosphate synthase; IspA, farnesyl diphosphate synthase; HepS, heptaprenyl diphosphate synthase component I; MenD, 2-oxoglutarate decarboxylase and 2-succinyl-5-enolpyruvyl-6-hydroxy-3-cyclohexene-1-carboxylic-acid synthase; MenB, 1,4-dihydroxy-2-naphthoyl-CoA synthase; MenA, 1,4-dihydroxy-2-naphthoate octaprenyltransferase; MenG, demethylmenaquinone methyltransferase.

As shown in Figure 6, MK-7 consists of a menadione parent ring and seven isoprene side chains. The main synthetic pathways included the Embden-Meyerhof pathway (EMP), glycerol metabolism pathway, pentose phosphate pathway (HMP), 2-C-methyl-D-erythritol-4-phosphate (MEP) pathway, and menaquinone (MK) synthesis pathway (Bentley and Meganathan, 1982; Ren et al., 2020). Comparison of the transcriptome results showed that alkali stress greatly influenced these pathways (Supplementary Table 1).

In glycerol metabolism and the EMP pathway, glycerol kinase (Glpk) was up-regulated 1.85-fold after alkali stress, which promoted glycerol consumption and utilization. Glyceraldehyde-3-phosphate dehydrogenase, enolase (Eno), and pyruvate kinase (PYK) were up-regulated by 1.16-, 0.92-, and 1.15-fold, respectively, and the EMP pathway flux increased. This result was consistent with that of Cheng et al. (2020), in which the expressions of Glpk, fructose bisphosphate aldolase (FBA), and phosphofructokinase (PFK) were up-regulated by 1.61, 0.91, and 2.31-fold, respectively, and the consumption of glycerol substrate increased. Eno and PYK were also up-regulated by a factor of 2.34 and 1.54, respectively, converting glyceraldehyde-3-phosphate to pyruvate, ultimately increasing MK-7 production (Cheng et al., 2020). Pyruvate is converted to acetyl-CoA by pyruvate dehydrogenases: dihydrolipoamide dehydrogenase (AcoL and AcoC). AcoL, AcoC, and citrate synthase (CS) were up-regulated by 6.08-, 5.70-, and 1.84-fold, respectively, promoting the accumulation of the important precursor acetyl-CoA. The up-regulation of these enzymes was consistent with previous research results, that is, the expression of Eno and PYK were conducive to the accumulation of more pyruvate and the formation of acetyl-CoA, strengthening the metabolic flux of the tricarboxylic acid (TCA) cycle (Cheng et al., 2020), thus accumulating more pyruvate and generating more isoprene side chains (Xu et al., 2012). The enhancement of glycerin metabolism and the EMP pathway can provide higher energy for cell growth and produce more precursors for MK-7 biosynthesis (Ma X. et al., 2019).

In the process of entering the MEP pathway, 1-deoxyxylulose-5-phosphate synthase (Dxs), the first level of rate-limiting enzyme of the MEP pathway, was found to be up-regulated 2.21-fold. The enzymes 1-deoxy-D-xylulose 5-phosphate reductoisomerase (Dxr), 2-C-methyl-D-erythritol 4-phosphate cytidyltransferase (IspD), 2-C-methyl-D-erythritol-2,4-cyclodiphosphate synthase (IspF), 1-hydroxy-2-methyl-2-(E)-butenyl 4-diphosphate reductase (IspH), farnesyl diphosphate synthase (IspA), heptaprenyl diphosphate synthase component I (HepS), and heptaprenyl diphosphate synthase component II (HepT) were up-regulated 1.05-, 0.24-, 0.72-, 0.65-, 0.32-, 2.27-, and 0.56-fold, respectively. The enzymes Dxs and Dxr have been consistently identified as rate-limiting enzymes in the MEP pathway (Lv et al., 2013). Yang et al. (2019) showed that the over-expression of *dxs* and *dxr* genes in the EMP pathway increased the fermentation yield of MK-7 to 69.50 mg/L. In addition, these authors proved through genetic engineering that the over-expression of *dxs*, *dxr*, *ispD*, and *ispF* genes in the MEP pathway could increase the yield of MK-7 (Yang et al., 2020). Xu and Zhang (2017) studied a strain of *Bacillus amylolytica* Y-2 and found that *hepS* gene encoding heptapreny pyrophosphate synthase could make MK-7 expression higher than other enzymes, which also provided information on the rate-limiting steps of different MK-7 producing bacteria. The expression of 4-hydroxy-3-methylbut-2-en-1-yl diphosphate synthase (IspG) was reduced by 1.11-fold. The over-expression of IspG might have increased the content of β-carotene, a product of other pathways in the metabolic pathway, which may have inhibited MK-7 production (Yang and Guo, 2014). The enzyme 4-diphosphocytidyl-2-C-methyl-D-erythritol kinase (IspE) was down-regulated 1.53-fold, possibly because the expression intensity of the *ispE* gene is negatively correlated with MK-7 synthesis (Yu et al., 1995).

Erythrose 4-phosphate forms shikimate through synthesis, dehydration and dehydrogenation, and finally reacts to form chorismate, which enters the MK pathway. The genes *menF*, *D*, *H*, *C*, *E*, and *B* encode six key enzymes and hydrolases to form the quinone skeleton 1,4-dihydroxy-2-naphthoate, followed by decarboxylation and methylation with heptaprenyl diphosphate formed by the MEP pathway under the action of the transferases 1,4-dihydroxy-2-naphthoate octaprenyltransferase (MenA) and demethylmenaquinone methyltransferase (MenG) to form menaphthoquinone. In the MK pathway, except for the down-regulated expression level of 1,4-dihydroxy-2-naphthoyl-CoA synthase (MenB) and O-succinylbenzoate-CoA synthase (MenC), the other 6 genes were up-regulated. Menaquinone-specific isochorismate synthase (MenF), 2-succinyl-5-enolpyruvyl-6-hydroxy-3-cyclohexene-1-carboxylic-acid synthase (MenD), 2-succinyl-6-hydroxy-2,4-cyclohexadiene-1-carboxylate synthase (MenH), O-succinylbenzoic acid-CoA ligase (MenE), MenA, and MenG expression were increased 0.75-, 2.64-, 0.85-, 0.47-, 3.08-, and 4.53-fold, respectively. It has been reported that MenD is the only enzyme that adds the intermediate thiamine pyrophosphate to the secondary substrate β-C (Dawson et al., 2008), the 1,4-dihydroxy-2-naphthoate octaprenyltransferase encoded by *menA*, polyisoprene pyrophosphate is used as the side chain to catalyze the formation of the water-soluble naphthalene compound 1,4-dihydroxy-2-naphthonic acid into membrane-bound 2-demethyl-menthoquinone, the precursor of MK (Suvarna et al., 1998; Hu et al., 2020). The results showed that enhancing the precursor supply and eliminating the by-product synthesis pathway were common methods to improve strain performance. MK-8 was the main menaquinone synthesized by *E. coli* (Kong and Lee, 2011). Over-expression of the menA or MenD, key enzymes associated with the supply of two precursors, increased the MK-8 content five times (Kong and Lee, 2011). Ma X. et al. (2019) found that over-expression of MenA in *Bacillus subtilis* increased MK-7 production three times. Using modular metabolic engineering design, Yang et al. (2019) also found that over-expression of the *menA* gene could increase the yield of MK-7 to 2.10 times that of the original strain *B. subtilis* 168. The over-expression of *menG* also increased the yield of MK-7 1.41 times by *Elizabethkingia meningoseptica* (Liu et al., 2018). These results might provide the impetus for further research on the transcriptional effects on MK-7 biosynthesis by *B. subtilis natto*.

## Conclusion

This study systematically analyzed the effect of stress adaptation and gene expression on MK-7 biosynthesis by *B. subtilis natto* under alkali stress conditions. Our analysis identified 820 DEGs, of which 608 genes up-regulated and 212 genes down-regulated. Data analysis results showed that the glucose metabolic pathway and MK pathway associated with MK-7 synthesis were enhanced, which promoted the accumulation of acetyl CoA, an important precursor, and the formation of an isoprene side chain, thereby promoting the synthesis and accumulation of MK-7. This study’s results provide a basis for improving the yield of MK-7 by *B. subtilis natto* under abiotic stress and valuable information to understand the molecular mechanism of MK-7 biosynthesis under environmental stress. In the future, the obtained *B. subtilis natto* with high MK-7 production will be applied to the production of MK-7-rich natto and other fermented products, which will also be important for health by preventing cardiovascular diseases, osteoporosis, cancer and nerve damage when consumed by people.

## Data Availability Statement

The original data on RNA sequencing have been uploaded to the NCBI Sequence Read Archive (SRA) under accession number PRJNA818476.

## Author Contributions

XC and LL contributed to conception and design of the study. CSh, HZ, and CSu performed the statistical analysis. XC wrote the first draft of the manuscript. CSh, HZ, CL, GZ, AL, and PD wrote sections of the manuscript. All authors contributed to manuscript revision, read, and approved the submitted version.

## Conflict of Interest

The authors declare that the research was conducted in the absence of any commercial or financial relationships that could be construed as a potential conflict of interest.

## Publisher’s Note

All claims expressed in this article are solely those of the authors and do not necessarily represent those of their affiliated organizations, or those of the publisher, the editors and the reviewers. Any product that may be evaluated in this article, or claim that may be made by its manufacturer, is not guaranteed or endorsed by the publisher.
