# Supplementary figures and images for: Effects of Alkali Stress on the Growth and Menaquinone-7 Metabolism of Bacillus subtilis natto
Source: Front Microbiol. 2022 Apr 28;13:899802. doi: 10.3389/fmicb.2022.899802 (PMC9096614; doi:10.3389/fmicb.2022.899802)

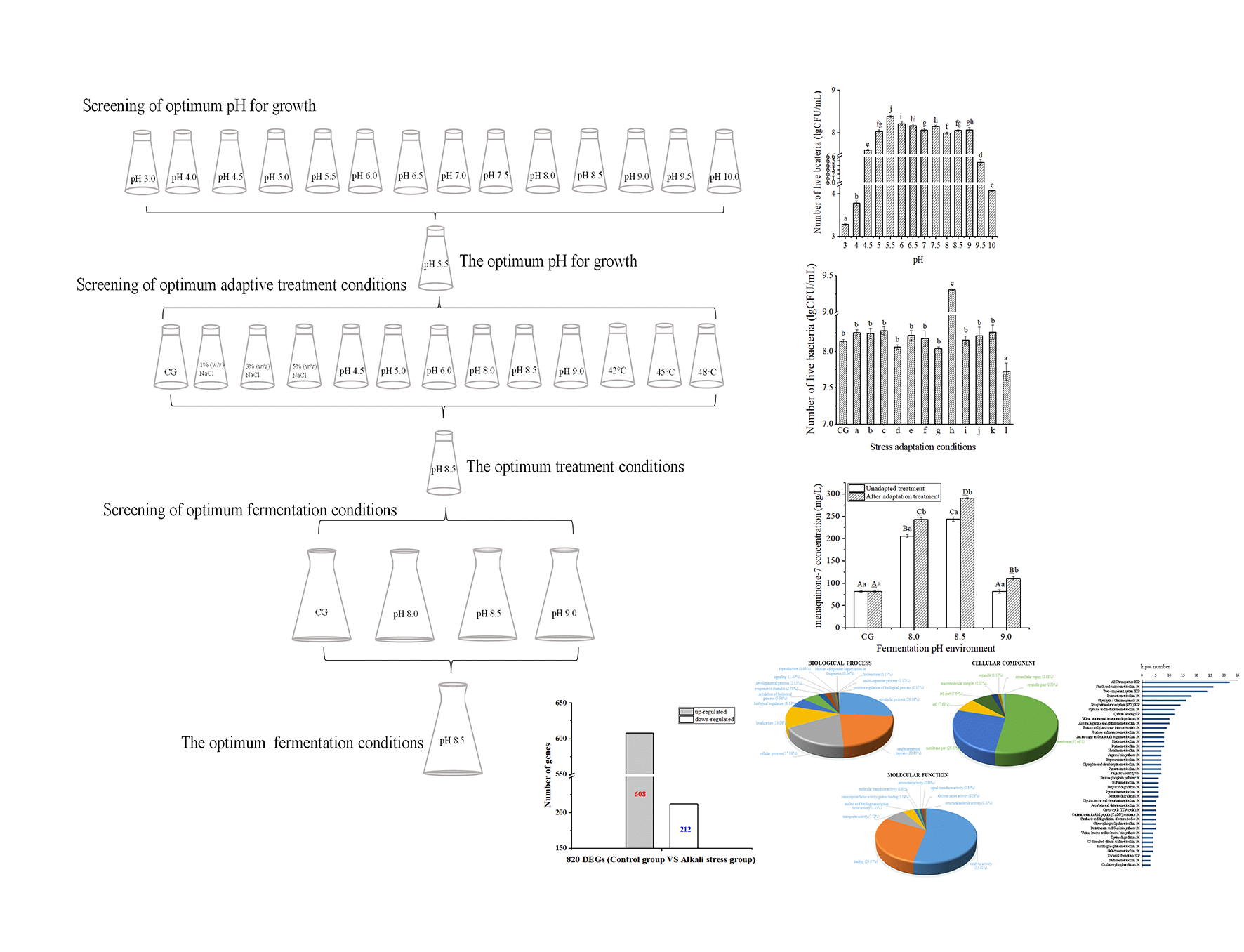

Supplement: Supplementary file 1 [file Image_1.TIF]
