# Supplementary material for: Effects of Alkali Stress on the Growth and Menaquinone-7 Metabolism of Bacillus subtilis natto
Source: Front Microbiol. 2022 Apr 28;13:899802. doi: 10.3389/fmicb.2022.899802 (PMC9096614; doi:10.3389/fmicb.2022.899802)
Supplement: Supplementary file 2 [file Table_1.DOCX]

Supplementary Material

Tab. S1 Key enzymes in MK-7 synthesis pathway

| Gene | Enzyme  abbreviation | Gene ID | Enzyme name | Fold-change |
| --- | --- | --- | --- | --- |
| *glpk* | GlpK | BSU_09290 | glycerol kinase | 1.85 |
| *gapB* | GapB | BSU_29020 | glyceraldehyde-3-phosphate dehydrogenase | 1.16 |
| *eno* | Eno | BSU_33900 | enolase | 0.92 |
| *pyk* | PYK | BSU_29180 | pyruvate kinase | 1.15 |
| *citA* | CS | BSU_09440 | citrate synthase | 1.84 |
| *acoL* | AcoL | BSU_08090 | acetoin dehydrogenase E3 component (dihydrolipoamide dehydrogenase) | 6.08 |
| *acoC* | AcoC | BSU_08080 | acetoin dehydrogenase E2 component (dihydrolipoamide acetyltransferase) | 5.70 |
| *dxs* | Dxs | BSU-24270 | 1-deoxyxylulose-5-phosphate synthase | 2.21 |
| *dxr* | Dxr | BSU_16550 | 1-deoxy-D-xylulose 5-phosphate reductoisomerase | 1.05 |
| *ispD* | IspD | BSU_00900 | 2-C-methyl-D-erythritol 4-phosphate cytidylyltransferase | 0.24 |
| *ispE* | IspE | BSU_00460 | 4-diphosphocytidyl-2-C-methyl-D-erythritol kinase | -1.53 |
| *ispF* | IspF | BSU_00910 | 2-C-methyl-D-erythritol-2,4-cyclodiphosphate synthase | 0.72 |
| *ispH* | IspH | BSU_25160 | 1-hydroxy-2-methyl-2-(E)-butenyl 4-diphosphate reductase | 0.65 |
| *ispG* | IspG | BSU_25070 | 4-hydroxy-3-methylbut-2-en-1-yl diphosphate synthase | -1.11 |
| *ispA* | IspA | BSU_24280 | farnesyl diphosphate synthase | 0.32 |
| *hepS* | HepS | BSU_22760 | heptaprenyl diphosphate synthase component I | 2.27 |
| *hepT* | HepT | BSU_22740 | heptaprenyl diphosphate synthase component II | 0.56 |
| *menF* | MenF | BSU_30830 | menaquinone-specific isochorismate synthase | 0.75 |
| *menD* | MenD | BSU_30820 | 2-succinyl-5-enolpyruvyl-6-hydroxy-3-cyclohexene-1-carboxylic-acid synthase | 2.64 |
| *menH* | MenH | BSU_30810 | 2-succinyl-6-hydroxy-2,4-cyclohexadiene-1-carboxylate synthase | 0.85 |
| *menC* | MenC | BSU_30780 | O-succinylbenzoate-CoA synthase | -0.63 |
| *menE* | MenE | BSU_30790 | O-succinylbenzoic acid-CoA ligase | 0.47 |
| *menB* | MenB | BSU_30800 | 1,4-dihydroxy-2-naphthoyl-CoA synthase | -1.26 |
| *menA* | MenA | BSU_38490 | 1,4-dihydroxy-2-naphthoate octaprenyltransferase | 3.08 |
| *menG* | MenG | BSU_22750 | demethylmenaquinone methyltransferase | 4.53 |

The original data on RNA sequencing has been uploaded to the public database (PRJNA818476).
